# A new record linkage for assessing infant mortality rates in Ontario, Canada

**DOI:** 10.17269/s41997-019-00265-6

**Published:** 2019-12-19

**Authors:** Deshayne B. Fell, Alison L. Park, Ann E. Sprague, Nehal Islam, Joel G. Ray

**Affiliations:** 1 University of Ottawa, Ottawa, Ontario Canada; 2 ICES, Ontario, Canada; 3 Children’s Hospital of Eastern Ontario (CHEO) Research Institute, Centre for Practice Changing Research, 401 Smyth Road, Room L-1154, Ottawa, Ontario K1H 8L1 Canada; 4 Better Outcomes Registry & Network, Ottawa, Ontario Canada; 5 Departments of Medicine, Health Policy Management and Evaluation, and Obstetrics and Gynecology, St. Michael’s Hospital, University of Toronto, Toronto, Canada

**Keywords:** Infant mortality, Newborn health, Data linkage, Mortalité infantile, Santé des nouveau-nés, Couplage de données

## Abstract

**Objective:**

Infant mortality statistics for Canada have routinely omitted Ontario—Canada’s most populous province—as a high proportion of Vital Statistics infant death registrations could not be linked with their corresponding Vital Statistics live birth registrations. We assessed the feasibility of linking an alternative source of live birth information with infant death registrations.

**Methods:**

All infant deaths occurring before 365 days of age registered in Ontario’s Vital Statistics in 2010–2011 were linked with birth records in the Canadian Institute for Health Information’s hospitalization database. Crude birthweight-specific and gestational age-specific infant mortality rates were calculated, and rates examined according to maternal and infant characteristics.

**Results:**

Of 1311 infant death registrations, only 47 (3.6%) could not be linked to a hospital birth record. The overall crude infant mortality rate was 4.7 deaths per 1000 live births (95% CI, 4.4 to 4.9), the same as previously reported for the rest of Canada in 2011. Infant mortality was higher in women < 20 years (5.8 per 1000 live births) and ≥ 40 years (5.9 per 1000 live births), and lowest among those aged 25–29 years (3.9 per 1000 live births). Infant mortality was notably higher in the lowest (5.1 per 1000 live births) residential income quintile than the highest (3.4 per 1000 live births).

**Conclusion:**

Use of birth hospitalization records resulted in near-complete linkage of all Vital Statistics infant death registrations. This approach could enhance the conduct of representative surveillance and research on infant mortality when direct linkage of live birth and infant death registrations is not achievable.

**Electronic supplementary material:**

The online version of this article (10.17269/s41997-019-00265-6) contains supplementary material, which is available to authorized users.

## Résumé

**Objectif:**

Les statistiques sur la mortalité infantile au Canada omettent couramment l’Ontario, pourtant la province la plus populeuse du pays, car une importante proportion des enregistrements de décès de nourrissons dans les statistiques de l’état civil ne peut pas être couplée avec les enregistrements correspondants de naissances d’enfants vivants dans ces mêmes statistiques. Nous avons évalué la faisabilité de coupler une autre source d’informations sur les naissances d’enfants vivants avec les enregistrements de décès de nourrissons.

**Méthode:**

Tous les décès de nourrissons survenus avant l’âge de 365 jours enregistrés dans les statistiques de l’état civil de l’Ontario en 2010-2011 ont été couplés aux dossiers de naissances dans la base de données sur les hospitalisations de l’Institut canadien d’information sur la santé. Les taux de mortalité infantile bruts, spécifiques au poids de naissance et spécifiques à l’âge gestationnel ont été calculés, puis examinés à la lumière des caractéristiques des mères et des nourrissons.

**Résultats:**

Sur 1 311 enregistrements de décès de nourrissons, seulement 47 (3,6 %) n’ont pas pu être couplés à un dossier de naissance à l’hôpital. Le taux brut global de mortalité infantile était de 4,7 décès pour 1 000 naissances d’enfants vivants (IC de 95 % : 4,4 à 4,9), le même que celui rapporté antérieurement pour le reste du Canada en 2011. La mortalité infantile était plus élevée chez les femmes de < 20 ans (5,8 pour 1 000 naissances d’enfants vivants) et de ≥ 40 ans (5,9 pour 1 000 naissances d’enfants vivants); elle était la plus faible chez les femmes de 25 à 29 ans (3,9 pour 1 000 naissances d’enfants vivants). La mortalité infantile était remarquablement plus élevée dans le quintile de revenu résidentiel inférieur (5,1 pour 1 000 naissances d’enfants vivants) que dans le quintile supérieur (3,4 pour 1 000 naissances d’enfants vivants).

**Conclusion:**

L’utilisation des dossiers des naissances à l’hôpital a permis un couplage presque complet des enregistrements de décès de nourrissons dans les statistiques de l'état civil. Cette technique pourrait améliorer la conduite d’activités de surveillance et de recherche représentatives sur la mortalité infantile quand le couplage direct des enregistrements de naissances d’enfants vivants et des enregistrements de décès de nourrissons n’est pas réalisable.

## Introduction

The infant mortality rate is an important population health indicator, reflecting the well-being of infants, children, pregnant women, and their families as well as the quality of health care (Public Health Agency of Canada 2008). For decades, the infant mortality rate has been a key metric of global initiatives to improve child health such as the United Nations’ Millennium Development Goals (United Nations 2015) and Sustainable Development Goals (United Nations Economic and Social Council 2017; You et al. 2015). Although data on infant mortality can be obtained from a variety of sources, the gold standard used by most high-income countries is a civil registration system that continuously records births and deaths, which are then reported to a centralized authority (Setel et al. 2007). Challenges with birth and death Vital Statistics data collection among low- and middle-income countries hinder progress toward reporting robust estimates of infant mortality (Setel et al. 2007).

In Ontario, Vital Statistics birth and death certificates are registered by the Office of the Registrar General within ServiceOntario in the Ministry of Government and Consumer Services. ServiceOntario regularly transfers these Vital Statistics registration files to Statistics Canada, where they are combined with registrations from other provinces and territories to create the national-level Vital Statistics Birth and Death Databases. Statistics Canada regularly performs a national-level record linkage of live birth registrations with infant death registrations to create a linked live birth–infant death file. This linked file is used extensively for national research and surveillance by the Canadian Perinatal Surveillance System (CPSS) within the Public Health Agency of Canada (Ananth et al. 2009; Deb-Rinker et al. 2015; Gilbert et al. 2013; Joseph et al. 2002, 2012; Public Health Agency of Canada 2008, 2017). A similar linkage of birth and infant death registrations is performed annually in the United States and the resulting linked file is a cornerstone of maternal and child health surveillance (Buehler et al. 2000).

In 2008, a surveillance report by the CPSS noted that a high proportion of Vital Statistics infant death registrations in Ontario could not be linked to their corresponding Vital Statistics live birth registration record (Public Health Agency of Canada 2008). This issue reached a peak in 2004, when 50% of infant death registrations in Ontario could not be linked to their Vital Statistics live birth registration, in stark contrast to the rest of Canada, where only 1% of infant death registrations remained unlinked. Throughout the 20-year period from 1991 to 2010, the proportion of unlinked infant death registrations in Ontario ranged from 19% to 50% (Figure S1). Although the reasons for this issue throughout the 1990s and early 2000s were not entirely clear, there is some indication that service fees charged to parents for live birth registration during that period (since rescinded) may have resulted in incomplete registrations of infants who later died. In addition, a majority of the unlinked infant deaths occurred early in the neonatal period and from causes such as short gestation, suggesting that these infants died following complicated pregnancies and birth and thus little impetus for grieving parents to complete birth paperwork (Public Health Agency of Canada 2008). And finally, an informal survey of perinatal programs in Canada in 2010 found that while most other provinces required that the parental component of the Vital Statistics birth registration be completed prior to hospital discharge regardless of the infant’s health, less than one third of Ontario hospitals reported discussing the need for birth registration with parents prior to leaving the hospital (unpublished data, BORN Ontario, 2010). When a Statement of Live Birth form (i.e., Form 2) is not received from parents by ServiceOntario, the birth registration is considered “incomplete” and is not included in the electronic file of Vital Statistics live birth data that are available for demographic and epidemiologic purposes (Woodward et al. 2003).

As the linkage of infant death registrations with information from the birth is essential in order to compute birthweight-specific and gestational age-specific infant mortality rates (Buehler et al. 2000; Joseph et al. 2012; Public Health Agency of Canada 2008), these data quality issues led to the systematic exclusion of Ontario from national perinatal surveillance reports and research studies that rely on Vital Statistics data (Public Health Agency of Canada 2008). Given that approximately 40% of all births in Canada occur in Ontario (Public Health Agency of Canada 2008), its exclusion from national perinatal statistics is an important gap. In this study, we assessed the feasibility of linking an alternative source of birth information with infant death registrations in order to report Ontario’s infant mortality rate.

## Methods

### Study design and data sources

This study was conducted at ICES—a not-for-profit provincial research entity that houses a large network of health administrative databases (https://www.ices.on.ca/). We conducted a retrospective cohort study by linking all 2010 and 2011 Ontario Vital Statistics infant death registrations (i.e., death registrations with an age at death of < 365 days after birth) with 2009 to 2011 hospital birth records recorded within the Canadian Institute for Health Information’s Discharge Abstract Database (CIHI-DAD). Infant death registrations in Ontario contain information on the infant’s age at the time of death and underlying cause of death, as well as some limited demographic information. ServiceOntario regularly transfers the provincial death registration files to ICES, where they can be securely linked with other provincial health administrative databases. The CIHI-DAD, also held at ICES, is a health care administrative database containing hospital separation abstracts from all acute care hospitalizations. Each abstract contains demographic information (e.g., age, postal code of residence, and vital status at the time of hospital separation) and medical diagnosis codes (primary diagnosis and up to 24 additional diagnoses), as well as other data elements, including gestational age for childbirth-related hospitalizations. Hospitalizations resulting in a birth generate both a maternal and a newborn/stillbirth abstract. The ICES-derived MOMBABY database contains linked CIHI-DAD maternal and newborn hospital birth abstracts, as well as unlinked maternal and newborn birth abstracts (henceforth known as orphan birth records). The Registered Persons Database (RPDB) is a population-based registry which provided additional information, such as postal code, which enabled us to link study data with the 2006 Canadian Census to obtain information on neighbourhood income and rural residence.

### Data linkage

These datasets were linked using unique encoded identifiers and analyzed at ICES. We used both deterministic and probabilistic methods to link infant death registrations from the calendar years 2010 and 2011 with hospital birth records from 2009 to 2011 to create a “period linked file” (Mathews and MacDorman 2011) (Figure S2). We first attempted to deterministically link all infant death registrations to birth records in CIHI-DAD/MOMBABY using unique encoded identifiers where available. We then probabilistically linked remaining infant death registrations using attributes common to both files: infant date of birth, place of death, infant sex, and residential postal code. The reference file for the record linkage comprised births from Jan. 1, 2009 to Dec. 31, 2011, extracted from the CIHI-DAD/MOMBABY databases. For the reference file, newborn (linked and orphan birth records) and maternal stillbirth records were also included in the event that any record classified as an infant death in the Vital Statistics death registrations was classified as a stillbirth in CIHI-DAD/MOMBABY. This type of variation, while infrequent, is known to occur for live births and stillbirths around the borderline of viability (i.e., infants born extremely preterm or at very low birthweight) (Ehrenthal et al. 2011; Joseph et al. 2012, 2015).

The study dataset is securely stored at ICES in coded form. While data sharing agreements prohibit ICES from making the dataset publicly available, access may be granted to those who meet pre-specified criteria for confidential access (available at www.ices.on.ca/DAS). The full dataset creation plan and underlying analytic code are available from the authors upon request. This study was approved by the Children’s Hospital of Eastern Ontario Research Ethics Board and ICES Privacy Office. The use of data in this project was authorized under section 45 of Ontario’s Personal Health Information Protection Act.

### Analyses

First, we determined the number and proportion of all infant death registrations that could not be linked to their corresponding hospital birth record. We expected a small number of infant death registrations to remain unlinked to a birth record in the reference file for three reasons: (i) the CIHI-DAD database only collects data on births weighing ≥ 500 g or occurring at ≥ 20 weeks’ gestation; thus, births not meeting either of these thresholds (all of whom would have a poor prognosis) would not be included in the CIHI-DAD or MOMBABY database; (ii) the birth took place in another province or outside a hospital setting from which the CIHI-DAD does not collect data (e.g., home births); and (iii) the infant death was registered following a termination of pregnancy beyond 20 weeks of gestation that resulted in a live birth, which may not be consistently captured by the CIHI-DAD database. Since we expected the infant death registrations that did not link with a hospital birth record (unlinked deaths) to differ from those that did link (linked deaths), we compared the timing, cause, and location of infant death between these two groups using chi-square tests for categorical variables and Mann–Whitney tests for continuous variables.

We then computed crude rates of neonatal (0 to 27 days) and overall infant mortality (0 to 364 days) per 1000 live births, as well as post-neonatal (28 to 364 days) mortality rates per 1000 neonatal survivors (i.e., all infants still alive by 28 days following birth; see Table S1 for definitions). Among the linked infant death registrations, we also computed birthweight-specific mortality rates restricted to all births ≥ 500 g and gestational age–specific mortality rates within several categories of preterm gestational ages, where numbers permitted (i.e., where the numerator was greater than 5). We generated 95% confidence intervals (CI) for all rates using the exact binomial method. Where possible, our mortality rates were compared with existing publicly reported rates for the rest of Canada from the same time period (Public Health Agency of Canada 2017). We additionally analyzed infant mortality rates by a variety of maternal and infant characteristics: maternal age group, rural versus urban residence, geographic region of residence (based on the Ontario health region, known as the Local Health Integration Network, where the mother resided), plurality, infant sex, and residential neighbourhood income quintile. Finally, we also computed crude infant mortality rates for modified international collaborative effort (ICE) cause of death groups (Public Health Agency of Canada 2008), where numbers permitted (i.e., > 5 infant deaths per group).

## Results

In the calendar years 2010 and 2011, there were 1311 infant deaths in the Vital Statistics death registrations. There were an additional 85 infant deaths recorded in the ICES RPDB file that were not registered with Vital Statistics, and these were not included in the analyses. Of the 1311 registered infant deaths, 1264 records (96.4%) were successfully linked with a birth record in the CIHI-DAD/MOMBABY databases. Compared with the linked infant death records, unlinked infant deaths were more likely to occur later—the median age at death among linked records was 1 day (interquartile range (IQR), 0–22) compared with 30 days (IQR, 1–61) in the unlinked records. The proportion of infants who died within the early neonatal period (0 to 6 days) was 64% among linked infant deaths compared with 34% among unlinked deaths. Conversely, a higher proportion of infant deaths occurred in the post-neonatal period and outside a hospital setting among unlinked deaths compared with linked deaths (Table 1). Overall, the crude infant mortality rate in Ontario for the 2010 and 2011 calendar years combined was 4.7 per 1000 live births (95% CI, 4.4 to 4.9; Table S2). With respect to timing of death, 77% (1008/1311) of infant deaths occurred in the neonatal period (0–27 days), yielding a crude neonatal mortality rate of 3.6 deaths per 1000 live births (95% CI, 3.4 to 3.8). The remaining 23% (303 cases) occurred in the post-neonatal period (28–364 days) for a rate of 1.1 per 1000 neonatal survivors (95% CI, 1.0 to 1.2).

**Table 1:** Characteristics of unlinked and linked infant death records. All data are shown as a number (%) unless otherwise indicated

Characteristic	Unlinked infant death records* (*n* = 47)	Linked infant death records (*n* = 1264)	*p* value
Calendar year of infant death			
2010	24 (51.1)	649 (51.3)	0.97
2011	23( 48.9)	615 (48.7)	
Median (IQR) age at death (days)	30 (1–61)	1 (0–22)	< 0.001
Death within 24 h			
No	37 (78.7)	614 (48.6)	< 0.001
Yes	10 (21.3)	650 (51.4)	
Early neonatal death at 0–6 days			
No	31 (66.0)	453 (35.8)	< 0.001
Yes	16 (34.0)	811 (64.2)	
Neonatal death at 0–27 days			
No	24 (51.1)	279 (22.1)	< 0.001
Yes	23 (48.9)	985 (77.9)	
Post-neonatal death at 28–364 days			
No	23 (48.9)	985 (77.9)	< 0.001
Yes	24 (51.1)	279 (22.1)	
Location of death			
Hospital	39 (83.0)	1216 (96.2)	< 0.001
Other	8 (17.0)	48 (3.8)	

*IQR* interquartile range

*Unlinked infant death records refer to those for which a corresponding live birth record could not be found in the CIHI-DAD/MOMBABY databases

Using the linked live birth–infant death file, and restricted to infants with a birthweight ≥ 500 g, the infant mortality rate was 3.3 per 1000 live births (95% CI, 3.1 to 3.5; Table 2). When restricted to those weighing ≥ 1000 g—the threshold recommended by the World Health Organization for international comparisons—the infant mortality rate was 2.0 per 1000 (95% CI, 1.9 to 2.2). There was a progressive decrease in infant mortality rates across increasing gestational age categories (Table 2).

**Table 2:** Birthweight-specific and gestational age-specific infant mortality rates for Ontario, Canada, 2010–2011

	No. of infant deaths	No. of live births	Infant mortality rate per 1000 livebirths (95% CI)
Birthweight (grams)			
< 500	331	375	882.7 (847.1–912.4)
500–999	363	1150	315.7 (289.3–343.0)
1000–1499	77	1683	45.8 (36.5–56.5)
1500–1999	71	3691	19.2 (15.2–24.1)
2000–2499	72	12,153	5.9 (4.7–7.4)
2500–2999	114	47,132	2.4 (2.0–2.9)
3000–3499	126	104,457	1.2 (1.0–1.4)
≥ 3500	109	110,283	1.0 (0.8–1.2)
≥ 500 g	932	280,549	3.3 (3.1–3.5)
≥ 1000 g	569	279,399	2.0 (1.9–2.2)
Gestational age at birth (weeks)			
< 25	595	745	798.7 (768.7–826.3)
25–29	139	1535	90.6 (77.0–105.7)
30–31	41	1324	31.0 (22.6–41.4)
32–33	41	2768	14.8 (10.8–19.9)
34–36	100	16,664	6.0 (4.9–7.3)
37–39	248	152,124	1.6 (1.4–1.8)
≥ 40	99	105,706	0.9 (0.8–1.1)

Among 1264 linked infant death registrations. One additional linked record was excluded due to missing information on birthweight and gestational age

**Fig. 1:**
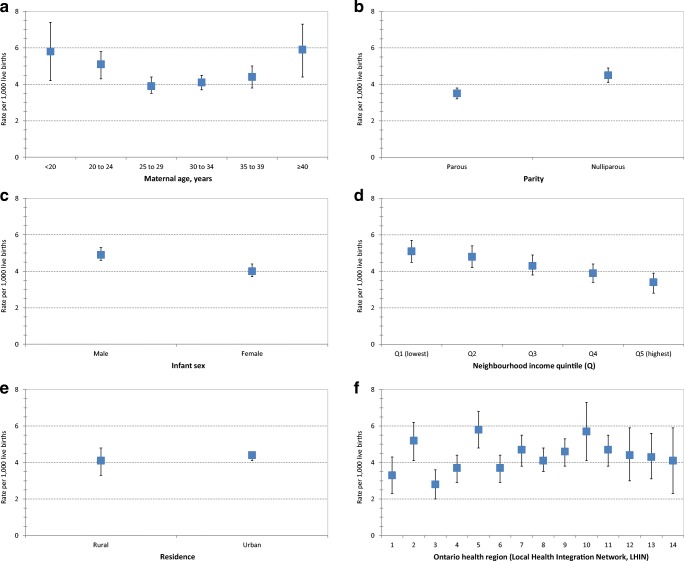
Infant mortality rates by maternal age (**a**), parity (**b**), infant sex (**c**), neighbourhood income quintile (**d**), residence (**e**), and Ontario health region (**f**). Data tables for these rates can be found in Table S3. The displayed rates were computed among the 1264 linked infant death registrations. Ontario health region was based on maternal residence in one of the Local Health Integration Network (LHIN) regions of Ontario

**Fig. 2:**
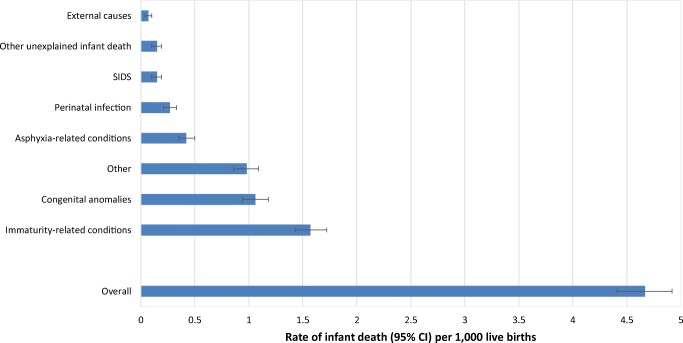
Cause-specific rates of infant mortality in Ontario, Canada, 2010–2011. Data tables for these rates can be found in Table S4. The displayed rates were computed among all 1311 infant death registrations (i.e., linked and unlinked deaths)

Male infants had a rate of 4.9 deaths per 1000 live births (95% CI, 4.6 to 5.3), slightly higher than that of female infants (4.0 per 1000 live births; 95% CI, 3.7 to 4.4) (Fig. 1; Table S3). Mortality rates were higher among infants born to nulliparous women (4.5 per 1000 live births; 95% CI, 4.1 to 4.9) than parous women (3.5 per 1000 live births; 95% CI, 3.2 to 3.8). There was a U-shaped pattern for mortality according to maternal age, with the lowest infant mortality rate observed among women 25–29 years of age (3.9 per 1000 live births; 95% CI, 3.5 to 4.4). Infant mortality showed an inverse relation to rising neighbourhood income quintile, with the highest rate in the lowest income quintile (quintile 1: 5.1 per 1000 live births, 95% CI 4.5 to 5.7) and the lowest rate in the highest quintile (quintile 5: 3.4 per 1000 live births, 95% CI 2.8 to 3.9) (Fig. 1; Table S3). Although infant mortality rates were similar in rural and urban residential settings, there was geographic variation across Ontario’s health regions, ranging from a low of 2.8 infant deaths per 1000 live births (95% CI, 2.0 to 3.6) to a high of 5.8 per 1000 (95% CI, 4.8 to 6.8) (Fig. 1, Table S3). According to modified ICE groupings, cause-specific rates were highest for immaturity-related conditions (1.57 per 1000 live births; 95% CI, 1.43 to 1.72), followed by congenital anomalies (1.06 per 1000; 95% CI, 0.94 to 1.18) (Fig. 2; Table S4).

## Discussion

This study assessed the feasibility of using provincial database linkages to establish a source of data for reporting and researching infant mortality in Ontario. For the calendar years 2010 and 2011, 96% of Ontario’s Vital Statistics infant death registrations were successfully linked with provincial hospital birth records—a much higher linkage rate than seen through prior attempts to link the infant death registrations with Vital Statistics live birth registrations. For instance, in 2010—the most recent year for which information is publicly available—only 68% of Ontario’s Vital Statistics infant death registrations could be linked with their corresponding Vital Statistics live birth registration (Figure S1).

Child mortality is considered an important indicator of population health worldwide. Since deaths of infants under 1 year of age account for approximately 88% of all deaths among children under the age of 5 years (You et al. 2015), the first year is a particularly vulnerable time period. Understanding underlying risk factors and distribution of infant deaths is valuable to public health practitioners and policy makers—yet, comprehensive population-based information on infant mortality in Ontario has been constrained by challenges linking Vital Statistics infant death registrations with a source of data with complete ascertainment of live births. These issues ultimately led to the systematic exclusion of Ontario data from indicators in national reports and research studies that rely on Vital Statistics data sources (Public Health Agency of Canada 2008), which has posed a gap in reporting at the national and provincial levels for many years.

Linkages of infant death registrations with information from the live birth, when successful, not only permit the calculation of gestational age- and/or birthweight-specific infant mortality rates, which are strongly recommended to mitigate the impact of temporal or geographic differences in birth registration practices (Joseph et al. 2012), but also enable assessment of variation and disparities in infant mortality rates by important demographic and clinical characteristics. According to our new linkage of Vital Statistics infant death registrations with provincial hospital birth records, we estimated Ontario’s crude infant mortality rate for 2010–2011 to be 4.7 deaths per 1000 live births, similar to the crude rates from the rest of Canada (excluding Ontario) during the same time period (5.1 per 1000 live births and 5.0 per 1000 live births in 2010 and 2011, respectively) (Public Health Agency of Canada 2017). The infant mortality rates for live births ≥ 500 g were also similar between our calculations for Ontario and from the rest of Canada (excluding Ontario) for 2010 (Ontario: 3.3 per 1000 live births ≥ 500 g; rest of Canada: 3.9 per 1000 live births ≥ 500 g). In Ontario, as in the rest of Canada (Public Health Agency of Canada 2017) and the USA (Mathews et al. 2015), the leading causes of infant death were due to immaturity-related conditions and congenital anomalies. Similar to findings from other surveillance reports (Mathews et al. 2015), we observed variability in infant mortality rates by infant sex (higher in males), parity (higher in nulliparous women), maternal age (U-shaped pattern), and neighbourhood income level (inverse gradient). In the future, extending the current linkage to include the province’s maternal-child registry (Better Outcomes Registry & Network (BORN) Ontario) would permit even more in-depth assessment of other risk factors for infant mortality in Ontario, including maternal smoking and pre-pregnancy body mass index, which are not available within CIHI-DAD/MOMBABY. Moreover, the ascertainment of live births in BORN Ontario also includes midwifery home births and birth centre births, providing a more complete denominator.

Despite the success of the database linkages we performed, there are several limitations that warrant mention. First, there were an additional 85 records in the ICES RPDB file classified as infant deaths that were not found in the Vital Statistics death registration file. We did not include these records in our analyses, as our objective was to link the registered infant deaths from Vital Statistics. Most of these 85 deaths (63/85; 74%) occurred within 24 h of the birth, the median birth weight was 788 g, and the median gestational age was 26 weeks. It is unclear whether some of these events that were classified as infant deaths in the RPDB file could have been erroneously registered as a stillbirth with Vital Statistics, since we did not have access to the Vital Statistics stillbirth registration file. Inconsistent designation of live birth followed by infant death versus stillbirth is known to occur around the borderline of viability (Ehrenthal et al. 2011; Joseph et al. 2012, 2015). It is also possible that some of these 85 deaths were among the 47 unlinked records in our original file of 1264 Vital Statistics infant death registrations. Although we were unable to include non-hospital births (e.g., home births or those that took place in a birth centre) in our linkage because no abstract is submitted to CIHI in such cases, women eligible to give birth in non-hospital locations are typically evaluated as having healthy, low-risk pregnancies and infant deaths in this population would be extremely rare. Moreover, only about 2% of all births in Canada take place outside a hospital setting (Public Health Agency of Canada 2009).

## Conclusion

In summary, we have used a new live birth–infant death linkage strategy to estimate infant mortality rates in Ontario. After many years of limited reporting of this important population health metric in Ontario, we are reassured of having similar rates to other jurisdictions in Canada. A major benefit of this linkage, if replicated in subsequent years, would be the improved ability to monitor and research infant mortality more contemporaneously at the provincial level, enhancing our understanding of disparities and allowing for targeted interventions where required to improve population health.

## Electronic supplementary materials

ESM 1 (DOCX 50 kb)
