# Supplementary material for: A new record linkage for assessing infant mortality rates in Ontario, Canada
Source: Can J Public Health. 2019 Dec 19;111(2):278–85. doi: 10.17269/s41997-019-00265-6 (PMC7109219; doi:10.17269/s41997-019-00265-6)
Supplement: Supplementary file 1 — (DOCX 50 kb) [file 41997_2019_265_MOESM1_ESM.docx]

**Supplementary material**

**Figure S1: Proportion of infant death registrations unlinked with their corresponding birth registration in the national Vital Statistics record linkage between 1991 and 2010 ***

* Data from the Canadian Perinatal Surveillance System, Public Health Agency of Canada

**Figure S2. Linkage strategy between live births and infant deaths**

**

**

**Table S1. Definitions of perinatal and infant mortality indicators using a period linked file**

| **Indicator** | |  |
| --- | --- | --- |
| Neonatal mortality | Definition | Number of infant deaths during the neonatal period (day 0 to day 27, inclusive, following live birth) per 1,000 live births. |
|  | Numerator | All neonatal deaths in a given calendar year, regardless of whether the birth occurs in the same calendar year or the preceding calendar year. |
|  | Denominator | All live births occurring in a given calendar year, plus any live births in the preceding calendar year that correspond to a neonatal death in the given calendar year. |
|  | Data sources | Numerator: Ontario Vital Statistics infant death registration file  Denominator: CIHI-DAD/MOMBABY |
|  | Example | For e.g., for the calendar year 2011, the numerator would comprise all neonatal deaths in 2011, while the denominator would comprise all live births in 2011, as well as any live births that took place in 2010 for which the infant died in 2011 during the neonatal period. |
| Post-neonatal mortality | Definition | Number of infant deaths during the post-neonatal period (day 28 to day 364, inclusive, following live birth) per 1,000 neonatal survivors*. |
|  | Numerator | All post-neonatal deaths in a given calendar year, regardless of whether the birth occurs in the same calendar year or the preceding calendar year. |
|  | Denominator | All live births occurring in a given calendar year, plus any live births in the preceding calendar year that correspond to a post-neonatal death in the given calendar year (only those live births that survived to 28 days post-birth). |
|  | Data sources | Numerator: Ontario Vital Statistics infant death registration file  Denominator: CIHI-DAD/MOMBABY |
|  | Example | For e.g., for the calendar year 2011, the numerator would comprise all post-neonatal deaths in 2011, while the denominator would comprise all live births in 2011, as well as any live births that took place in 2010 for which the infant died in 2011 during the post-neonatal period (only those live births that survived to 28 days post-birth). |
| Infant mortality | Definition | Number of infant deaths during the first year (day 0 to day 364, inclusive, following live birth) per 1,000 live births. |
|  | Numerator | All infant deaths in a given calendar year, regardless of whether the birth occurs in the same calendar year or the preceding calendar year. |
|  | Denominator | All live births occurring in a given calendar year, plus any live births in the preceding calendar year that correspond to an infant death in the given calendar year. |
|  | Data sources | Numerator: Ontario Vital Statistics infant death registration file  Denominator: CIHI-DAD/MOMBABY |
|  | Example | For e.g., for the calendar year 2011, the numerator would comprise all infant deaths in 2011, while the denominator would comprise all live births in 2011, as well as any live births that took place in 2010 for which the infant died in 2011. |

*Live born infants who survived to at least 28 days following birth.

**Table S2. Crude rates of infant mortality, neonatal mortality and post-neonatal mortality, Ontario, 2010-2011 *†**

| **Infant mortality**  (0-364 days) | | **Neonatal mortality**  (0-27 days) | | **Post-neonatal mortality**  (28-364 days) | |
| --- | --- | --- | --- | --- | --- |
| No.  infant deaths | Rate (95% CI) per 1,000 live births | No.  neonatal deaths | Rate (95% CI) per 1,000 live births | No.  post-neonatal deaths | Rate (95% CI) per 1,000 neonatal survivors |
| 1,311 | 4.7 (4.4–4.9) | 1,008 | 3.6 (3.4–3.8) | 303 | 1.1 (1.0–1.2) |

*Among all 1311 infant death registrations (i.e., linked and unlinked deaths).

**†**Definitions provided in Supplementary Table S1.

**Table S3. Infant mortality rates by maternal and newborn characteristics ***

|  | **No. of infant deaths** | **No. of live births** | **Infant mortality rate per 1,000 live births**  **(95% CI)** |
| --- | --- | --- | --- |
| Maternal age group (years) |  |  |  |
| < 20 | 52 | 8,984 | 5.8 (4.2–7.4) |
| 20 to 24 | 171 | 33,795 | 5.1 (4.3–5.8) |
| 25 to 29 | 295 | 75,017 | 3.9 (3.5–4.4) |
| 30 to 34 | 376 | 91,937 | 4.1 (3.7–4.5) |
| 35 to 39 | 214 | 48,671 | 4.4 (3.8–5.0) |
| ≥ 40 | 64 | 10,927 | 5.9 (4.4–7.3) |
| Missing | 45 | 11,598 | 3.9 (2.7–5.0) |
|  |  |  |  |
| Parity |  |  |  |
| Parous | 520 | 148,806 | 3.5 (3.2–3.8) |
| Nulliparous | 539 | 120,383 | 4.5 (4.1–4.9) |
| Missing | 158 | 11,740 | 13.5 (11.4–15.5) |
|  |  |  |  |
| Infant sex |  |  |  |
| Male | 713 | 143,989 | 5.0 (4.6–5.3) |
| Female | 551 | 136,940 | 4.0 (3.7–4.4) |
|  |  |  |  |
| Neighbourhood income quintile (Q) |  |  |  |
| Q1 (lowest) | 305 | 59,661 | 5.1 (4.5–5.7) |
| Q2 | 256 | 53,599 | 4.8 (4.2–5.4) |
| Q3 | 233 | 53,956 | 4.3 (3.8–4.9) |
| Q4 | 224 | 56,991 | 3.9 (3.4–4.4) |
| Q5 | 147 | 43,569 | 3.4 (2.8–3.9) |
| Missing | 52 | 13,153 | 4.0 (2.9–5.0) |
|  |  |  |  |
| Residence |  |  |  |
| Rural | 112 | 27,471 | 4.1 (3.3–4.8) |
| Urban | 1058 | 241,559 | 4.4 (4.1–4.6) |
| Missing | 47 | 11,899 | 3.9 (2.8–5.1) |
|  |  |  |  |
| Ontario health region of maternal residence † |  |  |  |
| 1 | 41 | 12,354 | 3.3 (2.3–4.3) |
| 2 | 98 | 18,977 | 5.2 (4.1–6.2) |
| 3 | 45 | 16,031 | 2.8 (2.0–3.6) |
| 4 | 96 | 26,208 | 3.7 (2.9–4.4) |
| 5 | 125 | 21,665 | 5.8 (4.8–6.8) |
| 6 | 87 | 23,656 | 3.7 (2.9–4.4) |
| 7 | 117 | 25,103 | 4.7 (3.8–5.5) |
| 8 | 151 | 36,606 | 4.1 (3.5–4.8) |
| 9 | 142 | 31,074 | 4.6 (3.8–5.3) |
| 10 | 47 | 8,271 | 5.7 (4.1–7.3) |
| 11 | 119 | 25,514 | 4.7 (3.8–5.5) |
| 12 | 36 | 8,092 | 4.4 (3.0–5.9) |
| 13 | 46 | 10,608 | 4.3 (3.1–5.6) |
| 14 | 20 | 4,871 | 4.1 (2.3–5.9) |
| Missing | 47 | 11,899 | 3.9 (2.8–5.1) |
|  |  |  |  |
| Plurality |  |  |  |
| Singleton pregnancy | 1036 | 270,342 | 3.8 (3.6–4.1) |
| Multifetal pregnancy | 228 | 10,587 | 21.5 (18.8–24.3) |

* Among 1264 linked infant death registrations.

† Region based on maternal residence in one of the Local Health Integration Network (LHIN) regions of Ontario.

**Table S4. Cause-specific rates of infant mortality, Ontario, 2010–2011, among 280,976 live births**

| **Cause according to modified international collaborative effort (ICE) classification ^*^** | **No. of infant deaths** | **Infant mortality rate per 1,000 live births**  **(95% CI)** |
| --- | --- | --- |
|  |  |  |
| Congenital anomalies | 298 | 1.06 (0.94–1.18) |
| Asphyxia-related conditions | 119 | 0.42 (0.35–0.50) |
| Immaturity-related conditions | 442 | 1.57 (1.43–1.72) |
| Perinatal infection | 76 | 0.27 (0.21–0.33) |
| SIDS | 41 | 0.15 (0.10–0.19) |
| Other unexplained infant death | 42 | 0.15 (0.10–0.19) |
| External causes | 19 | 0.07 (0.04–0.10) |
| Other | 274 | 0.98 (0.86–1.09) |
| TOTAL | 1,311 | 4.67 (4.41–4.92) |

* Among all 1311 infant death registrations (i.e., linked and unlinked deaths).
